# Myocardial ischemia: no supply/demand mismatch but reduced blood flow per beat

**DOI:** 10.1007/s00395-025-01157-2

**Published:** 2026-01-16

**Authors:** Gerd Heusch

**Affiliations:** Cardioprotection Unit, Institute for Pathophysiology, West German Heart and Vascular Center, University of Duisburg-Essen, Hufelandtsr. 55, 45122 Essen, Germany

**Keywords:** Coronary blood flow, Myocardial ischemia

## Abstract

The views on myocardial ischemia are changing—with an increasing focus on plaque vulnerability in acute coronary syndromes and more attention to the coronary microcirculation in chronic coronary syndromes. The unifying paradigm of supply/demand mismatch to characterize myocardial ischemia was developed from experiments in dogs with coronary occlusion and reperfusion and later used to also characterize myocardial ischemia from stress- and exercise-induced ischemia in settings of epicardial coronary stenoses. However, the supply/demand paradigm of myocardial ischemia has fundamental problems and appears not well suited to explain clinical scenarios with coronary microvascular dysfunction. Demand is an anthropocentric/hypothetical parameter which cannot be measured. In fact, in detailed and extensive experiments in dogs and pigs, regional myocardial contractile function which largely determines energy consumption, is reduced proportionately with the reduction of blood flow during ischemia—there is a perfusion–contraction match. When coronary blood flow is expressed in µl/g per cardiac cycle, the relationships of flow and function in ischemic myocardium at rest and during exercise are superimposable. Supporting the view that flow determines function, beta-blockade increases blood flow per cardiac cycle and in consequence also increases contractile function of the ischemic myocardium rather than reducing its hypothetical demand. In acute coronary syndromes, again supporting the pivotal role of coronary blood flow, the only way to salvage ischemic myocardium is restoration of blood flow, and all maneuvers to protect ischemic myocardium such as ischemic conditioning work only in conjunction with reperfusion.

The views on the pathophysiology of myocardial ischemia are changing [27, 30]. For acute coronary events, attention has shifted from coronary stenosis to plaque vulnerability [57]. For chronic coronary syndromes, the 2024 European Society of Cardiology guidelines still follow the intellectual footsteps of many eminent scientists [20], including Braunwald [5, 47], and use the supply/demand mismatch between coronary blood flow and contractile function as the unifying paradigm to explain the pathophysiology of myocardial ischemia [65]. However, several manifestations of chronic coronary syndromes, in particular those involving coronary microvascular dysfunction such as angina (subjective) or ischemia (objective) with non-obstructive coronary arteries (ANOCA/INOCA) [3, 34] and refractory angina after revascularization [11] are difficult to understand from a perspective of supply/demand mismatch, since angiographic coronary blood flow is often normal and angina can occur in the absence of increased heart rate or blood pressure.

This supply/demand paradigm is intuitive to explain the precipitation of myocardial ischemia in the presence of coronary stenosis and reduced coronary dilator reserve in situations such as acute stress, exercise, and pain which are associated with sympathetic activation. The supply/demand comes in various formats, e.g., in coronary blood flow over the rate-pressure-product [39] or the diastolic pressure time integral (for supply) over the systolic pressure time integral (for demand) [37], all of which use global rather than regional contractile function to estimate demand. However, this supply/demand paradigm is anthropocentric and hypothetical [26]. Energy or oxygen demand is not the same as energy or oxygen consumption, although the terms demand and consumption in the context of myocardial ischemia are often used synonymously. In fact, while consumption can be measured, demand is a virtual parameter. The intuitive equation of demand and consumption collapses when during sympathetic activation by stress, exercise, or pain they move into opposite directions, i.e., the hypothetical demand increases, but the real consumption of ischemic myocardium decreases. According to thermodynamic laws, no energy-dependent reaction occurs in the absence of that energy, and no mammalian cell (including the cardiomyocyte) can exert an energy-depending function in the absence of the required free energy change of ATP-hydrolysis. In consequence, as soon as the free energy change of ATP-hydrolysis falls below a certain threshold, the cardiomyocyte can no longer exert contractile function (which has the highest energy dependence and largely determines myocardial energy consumption) or maintain ionic gradients (which manifest in ECG changes, but become apparent later than contractile dysfunction) [17]. ATP synthesis in cardiomyocytes is largely dependent on mitochondrial oxidative phosphorylation, and this is in turn dependent on oxygen delivery by coronary blood flow as well to a minor extent on oxygen extraction which is already near maximal in the normal heart. In the absence of coronary blood flow, there is little buffer capacity by myoglobin-bound O_2_, creatine phosphate, and anaerobic glycolysis such that contractile function ceases within seconds after an acute stop of coronary blood flow [62]. Thus, a mismatch between energy supply and energy usage for the contractile function which the cardiomyocyte really exerts, i.e., its real energy expenditure, cannot and does not exist. The ischemic cardiomyocyte is injured solely by its limited energy supply which does not let it exert its normal, let alone increased contractile function. A mismatch between coronary blood flow and contractile function can only exist transiently for as long as the above energy buffers suffice. The time limits for such transient mismatch between coronary blood flow and contractile function depend on the severity of myocardial ischemia, i.e., are in the range of a few seconds  with coronary occlusion [62] or minutes during strenuous exercise-induced ischemia [15] but may be more prolonged with modest reductions in coronary blood flow as in short-term hibernating myocardium [49, 60].

The focus must be on the region of the heart which is eventually affected by ischemia, different from the original idea of supply/demand mismatch which was derived from a global view on the entire heart [26, 47]. To better understand the precipitation of acute regional myocardial ischemia by sympathetic activation in the presence of a coronary stenosis and reduced coronary dilator reserve, the focus must also be directed on a single cardiac cycle and the coronary blood flow to support such cardiac cycle. With the sympathetic activation of acute stress, there is tachycardia and the number of cardiac cycles per time frame (usually min) increases, whereas diastolic duration, i.e., the time interval during which coronary blood flow occurs, decreases. To maintain the blood flow per cardiac cycle constant, and this is mandatory to maintain the individual cardiac cycle’s contraction amplitude, the coronary blood flow per time frame must increase in proportion to heart rate [23]. Such an increase in coronary blood flow is mediated by metabolic coronary dilation. The mechanisms of metabolic coronary dilation are still under intense debate and may involve endothelial signals and certain potassium channels in vascular smooth muscle cells and/or pericytes [10, 46]. The increase in contractile function during sympathetic activation is matched by an increased flow per cardiac cycle. However, the trajectories of flow/beat vs. heart rate and contractile function in normal myocardium may vary depending on oxygen-carrying capacity and myocardial oxygen extraction. Above the autoregulatory range, increases in blood flow have no impact on contractile function [59].

In the presence of coronary stenosis, the dilator reserve of the post-stenotic region is recruited to maintain baseline coronary blood flow, and therefore, its potential for an increase in blood flow during sympathetic activation and tachycardia is limited, ultimately with severe coronary stenosis exhausted. With a moderate coronary stenosis, baseline coronary blood flow and function may still be normal and blood flow per time frame may still increase during stress and tachycardia, but blood flow per beat will decrease, and in consequence, the contraction amplitude of each cardiac cycle also decreases, i.e., there is post-stenotic regional contractile dysfunction despite increased blood flow per min because blood flow per beat is decreased [45]. The observed contractile dysfunction may be preceded by a short-lasting (2–3 min) increase in contractile function which is sustained by the above-detailed energy buffers (myoglobin-bound O_2_, creatine phosphate, anaerobic glycolysis). The inability of coronary dilator reserve to maintain contractile function and blood flow per beat is not only related to its recruitment at baseline in compensation for the stenosis but also to active post-stenotic coronary vasoconstriction through the alpha-adrenoceptor activation by the sympathetic transmitter norepinephrine [21, 31, 32]. A redistribution of coronary blood flow away from the post-stenotic myocardial region through collaterals to adjacent non-ischemic regions which still have dilator reserve as well as a transmural redistribution from the ischemic subendocardium which is exposed to greater extravascular compression by left ventricular cavity pressure to the subepicardium also contributes to the reduction of blood flow in the most severely ischemic myocardium [2]. Such redistribution of blood flow from ischemic to non-ischemic myocardium is the only way by which the contractile function and energy expenditure of the non-ischemic myocardium impact on the ischemic region, again through reduced blood flow. In conscious dogs with a chronic coronary stenosis but normal myocardial blood flow and contractile function at baseline, exercise induces ischemia which reaches a steady state of reduced myocardial blood flow and reduced contractile function within 2–3 min. In the ischemic myocardial region, blood flow and contractile function are linearly related, i.e., *proportionately* reduced and contradicting a mismatch between supply and demand. The quantitative relationship between coronary blood flow and regional contractile function may vary and depend on the experimental preparation and model [24, 25]. In conscious dog models with good autoregulation, the relationship may be flatter within the autoregulatory range and steeper at lower levels of coronary blood flow [7, 9, 64]. In acute open-chest experiments, the relationship between flow and function may be more linear since oxygen extraction may not adequately increase with reduction of coronary blood flow [8], possibly secondary to heterogeneity of capillary perfusion [53]. In the experiment, subendocardial blood flow has a greater impact on contractile function than transmural blood flow [7, 9, 14]. Even in the experiment, it may be technically difficult to determine regional myocardial oxygen extraction at the same spatial resolution as flow and function. The more linear relationship of flow and function may also better reflect the human situation where atherosclerosis affects the entire coronary circulation and its autoregulation.

Moreover, when post-stenotic myocardial blood flow is expressed not per min, but per beat, its relation to contractile function (which is usually expressed as systolic wall excursion in percent of end-diastolic dimension in a single cardiac cycle) during graded coronary stenosis at baseline is superimposable with that during exercise-induced ischemia (Fig. 1). These flow–function relations are thus independent of the hemodynamic situation [15], again contradicting a mismatch between supply and demand in the ischemic myocardial region. A normal blood flow is 8–10 µl/g per cardiac cycle in conscious dogs [26]. More recently, detailed analyses of coronary blood flow and regional myocardial contractile function in anesthetized open-chest pigs during ischemia without and with dobutamine challenge confirmed the notion of perfusion–contraction matching and identified a threshold of 5–7.5 µl/g per cardiac cycle below which ischemia with contractile dysfunction occurred [12]. Canty and Weil [8] have critically reviewed the experimental studies demonstrating perfusion–contraction matching and, while also refuting a supply/demand paradigm, proposed the intriguing refinement to use oxygen delivery (coronary blood flow x arterial oxygen content) per cardiac cycle and its matching to contractile function since oxygen delivery also considers for situations such as hypoxemia or anemia [16, 40]. Admittedly, the concept of a relatively fixed and universal coronary blood flow/beat or oxygen delivery/beat is derived from a number of prior studies but has not been rigorously and prospectively tested over a wide variety of experimental models and hemodynamic situation. In addition, there are no studies in humans with systematic variations in coronary blood flow and contractile function that would permit the assessment of flow–function relationships and provide evidence for a flow/beat or oxygen delivery/beat threshold below which ischemia develops.

**Fig. 1:**
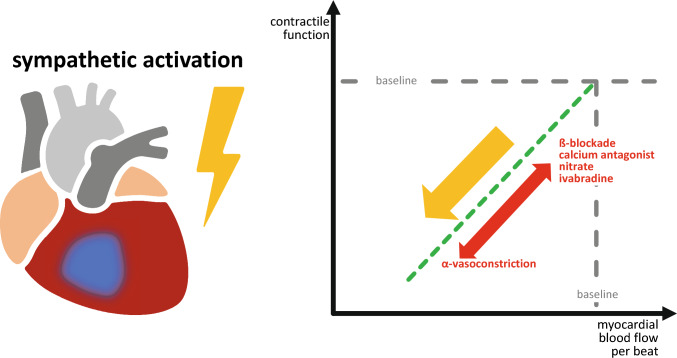
During exercise-induced myocardial ischemia, regional contractile function and myocardial blood flow per beat are matched, i.e., proportionately reduced in the ischemic region. Anti-ischemic/anti-anginal agents move flow per beat and function upwards along the proportionate flow–function relationship

Nevertheless, considerations of coronary blood flow per beat have therapeutic implications: beta-blockade [51], calcium antagonists such as diltiazem [50] or selective bradycardic agents [19, 36] attenuate exercise-induced tachycardia, thus increasing blood flow per beat and improving contractile function. The therapeutic action of beta-blockade is thus achieved by heart rate reduction and thus an increase of blood flow per cardiac cycle and not by reduced demand; in fact, beta-blockade even increases contractile function of the ischemic myocardium, whereas it decreases, as expected, the contractile function of normal myocardium [6, 18, 51]. If the reduction in heart rate by beta-blockade is prevented by pacing, alpha-adrenergic coronary vasoconstriction prevails and blood flow and contractile function per beat are reduced [18]. On the other hand, alpha blockade [31, 43] or a dilator calcium antagonist such as nifedipine [33] increase blood flow and contractile function per beat even at unchanged heart rate.

The match between perfusion and contraction has also become the cornerstone of hibernating myocardium, i.e., an adaptation of the myocardium to reduced blood flow by decreased contractile function [28, 29, 56] Sub-acutely over several hours, there may indeed be an adaptive downregulation of contractile function in response to reduced blood flow as long as there is some residual blood flow [49, 60]. Such adaptive downregulation may help to maintain myocardial viability in patients with an acute myocardial infarction such that reperfusion still rescues some viable myocardium after 24 h from symptom onset [52, 58]. Elegant studies by Canty et al. have, however, demonstrated that reduced contractile flow and function of more chronic hibernating myocardium develop in reverse mode, i.e., repetitive stress-induced ischemia with consequent stunning induces a prolonged state of reduced contractile function to which coronary blood flow adapts [13]. In any event, this hibernating myocardium is characterized by a perfusion–contraction match of each cardiac cycle and not a supply/demand mismatch, and contractile function may recover after revascularization. While there is solid support for perfusion–contraction matching from a number of studies using measures of coronary blood flow and regional contractile function in patients with hibernating myocardium [22, 35], its pathogenetic development in humans is not clear.

Incidentally, Braunwald’s proposal to understand myocardial ischemia as a supply/demand mismatch originated from dog experiments with acute coronary occlusion and infarct size as outcome [5, 47]. Clearly, infarct size is determined 1. by the affected perfusion territory of the occluded coronary artery, i.e., the area at risk, 2. by the duration of ischemia, and 3. the residual coronary blood flow, either antegrade or through collaterals. Demand per se has no role in infarct size development, and its hemodynamic determinants heart rate and contractile function, if anything, act again through reductions of residual blood flow [27]. Of note, the first placebo-controlled clinical trial to reduce “demand” in patients with acute myocardial infarction by intravenous propranolol came out neutral for indices of infarct size and clinical outcome, and this trial (MILIS) had Braunwald as last author [54]. While the MILIS trial on propranolol had been conducted before the reperfusion era, the reduction of infarct size by beta-blockade has remained contentious also for reperfused myocardial infarction [38, 42, 55]. It is therefore irritating and unfortunate that the fourth definition of myocardial infarction still embarks on the supply/demand mismatch paradigm for type II infarction when it just means to exclude plaque rupture or erosion as the cause of myocardial infarction.

In conclusion, I therefore advocate to abandon the supply/demand mismatch paradigm for acute and chronic coronary syndromes and to focus instead on the reduction of coronary blood flow per beat. Such a novel view on myocardial ischemia may open new avenues to better understand irritating issues in the arena of chronic coronary syndromes, such as ANOCA where epicardial atherosclerosis usually is present but insufficient to explain the initiation of ischemia [3, 34], persistent and refractory angina after revascularization [11], or the lack of difference in outcomes between optimal medical and interventional treatment [1, 4, 48]. In this respect, better, clinically relevant experimental models must be developed to study in particular the pathogenesis of ischemia from coronary microvascular dysfunction and to promote the development of novel, mechanistically founded therapies [34, 41, 61, 63]. The focus on coronary blood flow rather than on a hypothetical demand also strengthens the notion that all cardioprotective interventions to reduce infarct size in acute coronary syndromes require reperfusion, i.e., restoration of coronary blood flow [27, 30, 44]. Eventually, I advocate to go back to the original meaning of ischemia, as coined by Virchow [20], i.e., lack of blood (flow) with the implication that all mechanistically founded therapeutic interventions must increase coronary blood flow for any given cardiac cycle.
